# Three‐trocar tubeless natural orifice specimen extraction surgery in rectosigmoid cancer – a video vignette

**DOI:** 10.1111/codi.15081

**Published:** 2020-05-20

**Authors:** Y. Luo, P. Wang, X. Gu, J. Ye, J. Lin, M. Tan, P. T. Luo, J. T. Luo, M. Huang

**Affiliations:** ^1^ Department of Colorectal Surgery Guangdong Institute of Gastroenterology Guangdong Provincial Key Laboratory of Colorectal and Pelvic Floor Disease Supported by National Key Clinical Discipline The Sixth Affiliated Hospital Sun Yat‐sen University Guangzhou China; ^2^ Department of Surgery The People’s Hospital of Gaoming District Foshan China; ^3^ SEOX Financial Quotient (Guangzhou) Education Technology Ltd Guangzhou China; ^4^ Class 9 Grade 3 The Affiliated Foreign Language School of SCNU Guangzhou China; ^5^ Class 6 Grade 1 The Affiliated Foreign Language School of SCNU Guangzhou China

*Dear Editor,*

In this video (Video S1 in the online Supporting Information), we propose a modified natural orifice specimen extraction surgery (NOSES) technology, called three‐trocar tubeless NOSES, to radically resect rectosigmoid cancer; this procedure can avoid a large abdominal incision for extraction of the specimen. Only three trocar incisions are used to complete the whole procedure, and no tubes (e.g. nasogastric tube, drainage tube or catheter) are left *in situ* after surgery, which can minimize patient complications and pain.

Our patient was a 62‐year‐old man with a high rectal lesion shown on colonoscopy and pathologically confirmed as adenocarcinoma. The CT scan showed this to be localized in the upper rectum without distant metastasis.

We confirmed there was no liver or omental metastasis. Next, we dissected the lymph nodes and soft tissue along the inferior mesenteric artery. Meanwhile, we expanded the plane to free the sigmoid colon in order to stretch the colon easily later. After skeletonization of the distal rectum, we closed the rectal lumen at the distal margin by ligation. After an iodophors wash, we closed the proximal lumen by ligation and cut the bowel using a Harmonic Scalpel. A stapler anvil was placed into the abdominal cavity after separation of the bowel wall. Then, the specimen was amputated from the proximal colon and extracted from the anus. The distal lumen was closed using an Ethicon Endo GIA. Subsequently, we placed the stapler anvil into the proximal lumen and completed the anastomosis, and no leaks were found. The whole procedure was completed after closing the mesocolon and the trocar incisions.

## Conflicts of interest

The authors declare that they have no conflict of interest.

## Funding

This study was supported by the National Basic Research Program of China (973 Program) (no. 2015CB554001, JW), the National Natural Science Foundation of China (no. 81972245, YL; no. 81902877, HY), the Natural Science Fund for Distinguished Young Scholars of Guangdong Province (no. 2016A030306002, YL), the Tip‐top Scientific and Technical Innovative Youth Talents of Guangdong special support program (no. 2015TQ01R454, YL), Project 5010 of Clinical Medical Research of Sun Yat‐sen University‐5010 Cultivation Foundation (no. 2018026, YL), the Natural Science Foundation of Guangdong Province (no. 2016A030310222, HY; no. 2018A0303130303, HY), the Program of Introducing Talents of Discipline to Universities, and National Key Clinical Discipline (2012).

## Supporting information


**Video S1** **.** Three‐trocar tubeless natural orifice specimen extraction surgery in rectosigmoid cancer – a video vignette.

**Data S1.** Script of the video.

