# Supplementary material for: Three‐trocar tubeless natural orifice specimen extraction surgery in rectosigmoid cancer – a video vignette
Source: Colorectal Dis. 2020 May 20;22(10):1458. doi: 10.1111/codi.15081 (PMC7818471; doi:10.1111/codi.15081)
Supplement: Supplementary file 2 — Data S1. Script of the video. [file CODI-22-1458-s002.docx]

**Script of the video**

We are introducing a three-trocar Tubeless NOSES technology in rectosigmoid cancers. This is a 62-year-old male with a high rectal lesion shown in colonoscopy. Pathology confirmed that is an adenocarcinoma. CT scan shows this is a localized disease in the high rectum without distant metastasis. Here is the operation procedure. The abdominal cavity exploration finds there is no liver or omentum metastasis. The primary tumor locates in the high rectum. The first step is to move to the median area and dissect the lymph nodes and soft tissue along the inferior mesenteric artery (IMA). The best way to do this is to follow the plane between the Toldt’s fascia and mesocolon. Meanwhile, we expand the plane to free sigmoid colon, which can help us stretch the colon easily later. During this step, we need to identify and preserve the left ureter. After we skeletonized the distal rectum, we closed the rectal lumen at the distal margin by ligation. Then a surgeon will wash the distal rectum by iodophors carefully from the anus. Now we confirm the proximal margin of the resection. After the skeletonization, we close the proximal colon lumen by ligation. After a couple of times washing, the distal rectal lumen was clean. Then we cut the bowel by Harmonic Scalpel. After separating the bowel wall by a sterilized film, we place a stapler anvil into the abdominal cavity through rectal lumen from the anus. Then the specimen is amputated from the proximal colon and extracted from the anus then out of the body. Then we close the distal lumen by Ethicon Endo-GIA and wash the area a couple of times to minimize the potential contamination. Next, we place the stapler anvil into the proximal lumen and complete the anastomosis. The bubble testing shows there is no air leaking from the anastomosis. Then we close the mesocolon hiatal by suturing. And finally, we close the trocar incisions. Here the whole procedure was done and there’s no tube on patients after surgery. The duration of the surgery is about 90 minutes and the blood loss is less than 50 ml. The patients can take some liquid right after the surgery and walk around the following day. Bowel movement recover on the second day and the patient is discharged on the fifth day after surgery without complications. Thanks for your attention.
